# Gynæcological Diagnosis and the Microscope

**Published:** 1909-09-11

**Authors:** 


### Gynæcology.

### GYNÆCOLOGICAL DIAGNOSIS AND THE MICROSCOPE.

The accurate diagnosis of some of the diseases of women is often a matter of great difficulty or even impossibility by the ordinary means of inspection and palpation, and frequently the aid of the microscope must be called in to settle the question. This is especially the case in early carcinoma of the cervix or uterine body, in doubtful growths of the vulva, and in some large tumours of the uterus. The differential diagnosis of the many varieties of ovarian tumour can often be made only by the microscope, but this cannot be considered quite in the same category as early malignant growths of the uterus for instance. Ovarian tumours, with few exceptions, must be removed whether innocent or malignant, and the differentiation of them after removal is of scientific interest only. The removal of small pieces from the cervix or uterine body for diagnostic purposes is quite another matter, and upon accurate diagnosis turns the question of radical operations on the uterus.

There can be nothing more annoying to the medical man than to obtain, with great difficulty perhaps, a piece of the cervix, to send it to a pathologist, and finally obtain an indefinite report which leaves the question of malignancy in doubt. Yet this does happen occasionally, and the result is that opinions are freely expressed that the microscope is useless for the diagnosis of difficult gynæcological cases. It is often, however, not the fault of the microscope or the pathologist who uses it, but rather of the specimen itself which is to be examined. To obtain suitable specimens for examination is not an everyday occurrence in general practice, and the preservation of such specimens when obtained is not always clearly understood. In nothing is this seen so frequently as in specimens from the cervix and curetted fragments from the body of the uterus. It is not easy to cut a representative piece of tissue from a cervical growth, and it is practically impossible to get satisfactory curettings from the uterine body unless the cervix is fully dilated under an anæsthetic and a sharp curette used. For instance, it is impossible, as a rule, to get a satisfactory fragment from the uterus with, say, an ear spud or a tiny curette through the undilated cervix. Still worse, it is impossible as a rule to diagnose a malignant growth of the uterus from some vaginal discharge collected on a diaper and allowed to dry; and yet this is a common sample of the miracles expected of the pathologist. It is an absolute fact which cannot be controverted that a definite opinion can be given in at least 98 per cent. of cases provided the material is representative of the lesion and properly fixed and preserved.

In the case of a suspected growth of the os uteri a wedge of tissue must be *cut out*, so as to include a portion of the growth and the underlying tissues. It is useless to scrape a little *débris* off the surface or to shave off the surface with a knife. *The base of the growth must be included.* After removal the tissue must be *at once* placed in an efficient preservative, and not allowed either to dry hard in cotton wool or paper, or to macerate in water or saline solution. The simplest fixing fluid for general use is 70 per cent. alcohol, but one which is easy to make and very efficient is 10 per cent. of commercial formalin diluted with 90 per cent. of normal salt solution (.75 per cent.). Watery solutions of formalin without salt should never be used, for they so alter the tissues that they will not take a nuclear stain.

To remove a wedge from the os uteri, it must be exposed with a speculum in a good light, seized with a vulsellum, and then the required tissue cut out deliberately with knife and forceps. But the tissue removed must be representative of the lesion. Curetted fragments are more difficult to deal with, as they are so often mixed up with large quantities of blood. To get the best results the curettings should be carefully preserved until the operation is finished, and then floated in water or normal saline fluid. From this the organised tissues can be washed free of blood, picked out with forceps and dropped at once into the preserving fluid. If simply put into a bottle with blood, maceration always occurs, and glandular epithelium becomes shaken away from its attachments in a very short time. It does not matter how small the fragments are if they are picked out and preserved in this way: the pathologist can always cut good sections from them. When these tissues are properly fixed and hardened, sections must be cut from them by the paraffin method. Freezing methods may sometimes answer, but in general, where delicate glandular structures are concerned, paraffin sections are infinitely superior. Even if a rapid diagnosis is required, the paraffin method is the best of all, for in the case of small fragments like curettings, good sections can be obtained in about four hours. It is true that the freezing method can be used for *fresh* tissues, but the sections so obtained may not be sufficiently good to warrant a definite diagnosis.

The methods detailed above are equally efficacious for the diagnosis of materials left in the uterus after abortions or in cases of chorion epithelioma. It is true that special knowledge is required in dealing with gynæcological specimens; training in general pathology will not always suffice to help in the diagnosis of endometritis versus carcinoma, or retained conception products versus chorion epithelioma. It is practically hopeless for the medical man who has only the histological knowledge gained as a student to attempt the diagnosis of these conditions by the microscope, and it may be that the attempts of the unskilled have led to the distrust of the microscope which is so prevalent. It can only be repeated that to the skilled pathologist scarcely one case in fifty presents any difficulty provided the tissues are representative, properly preserved, and the sections from them cut by the paraffin method.

